# 5-Amino-1-phenyl-3-trifluoro­methyl-1*H*-pyrazole-4-carboxylic acid

**DOI:** 10.1107/S1600536809032188

**Published:** 2009-08-15

**Authors:** Francesco Caruso, Maria Valeria Raimondi, Giuseppe Daidone, Claudio Pettinari, Miriam Rossi

**Affiliations:** a Istituto Chimica Biomolecolare CNR, Ple Aldo Moro 5, 00185 Rome, Italy; b Dipartimento Chimica e Tecnologie Farmaceutiche, Universita di Palermo, Via Archirafi 32 90123, Palermo, Italy; c Dipartimento Chimica, Universita di Camerino, Via Sant’Agostino 1, 62032 MC, Italy; d Department of Chemistry, Vassar College, Poughkeepsie, NY 12604-0484, USA

## Abstract

In the title compound, C_11_H_8_F_3_N_3_O_2_, there are two mol­ecules in the asymmetric unit wherein the phenyl rings make dihedral angles of 65.3 (2) and 85.6 (2)° with the pyrazole rings. In the crystal, pairs of mol­ecules are held together by O—H⋯O hydrogen bonds between the carboxyl groups, forming a centrosymmetric dimer with an *R* ^2^_2_(8) motif. Intra­molecular N—H⋯O inter­actions are also present.

## Related literature

For general background, see: Caruso & Rossi (2004 ▶); Maggio *et al.*, (2008 ▶). For hydrogen-bond motifs, see: Bernstein *et al.* (1995 ▶).
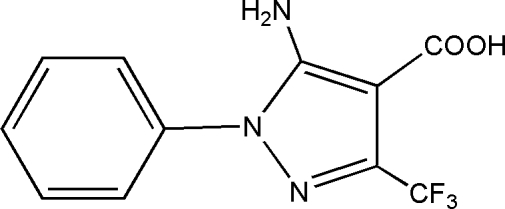


## Experimental

### 

#### Crystal data

C_11_H_8_F_3_N_3_O_2_
*M* *_r_* = 271.20
Monoclinic, 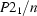

*a* = 9.757 (3) Å
*b* = 10.740 (3) Å
*c* = 21.277 (6) Å
β = 93.716 (3)°
*V* = 2225 (1) Å^3^
*Z* = 8
Mo *K*α radiation
μ = 0.15 mm^−1^
*T* = 125 K
0.30 × 0.18 × 0.05 mm

#### Data collection

Bruker APEXII CCD diffractometer
Absorption correction: multi-scan (*SADABS*; Bruker, 1997 ▶) *T*_min_ = 0.957, *T*_max_ = 0.993
20692 measured reflections
3772 independent reflections
2737 reflections with *I* > 2σ(*I*)
*R*_int_ = 0.072

#### Refinement

*R*[*F* ^2^ > 2σ(*F* ^2^)] = 0.051
*wR*(*F* ^2^) = 0.140
*S* = 1.01
3772 reflections
368 parameters
H atoms treated by a mixture of independent and constrained refinement
Δρ_max_ = 0.43 e Å^−3^
Δρ_min_ = −0.32 e Å^−3^

### 

Data collection: *SMART* (Bruker, 1997 ▶); cell refinement: *SAINT* (Bruker, 1997 ▶); data reduction: *SAINT*; program(s) used to solve structure: *SHELXS97* (Sheldrick, 2008 ▶); program(s) used to refine structure: *SHELXL97* (Sheldrick, 2008 ▶); molecular graphics: *PLATON* (Spek, 2009 ▶); software used to prepare material for publication: *SHELXL97*.

## Supplementary Material

Crystal structure: contains datablocks I, global. DOI: 10.1107/S1600536809032188/bx2218sup1.cif

Structure factors: contains datablocks I. DOI: 10.1107/S1600536809032188/bx2218Isup2.hkl

Additional supplementary materials:  crystallographic information; 3D view; checkCIF report

## Figures and Tables

**Table 1:** Hydrogen-bond geometry (Å, °)

*D*—H⋯*A*	*D*—H	H⋯*A*	*D*⋯*A*	*D*—H⋯*A*
N53—H531⋯O52	0.85 (4)	2.20 (4)	2.808 (3)	128 (3)
O51—H51⋯O2^i^	0.94 (4)	1.75 (4)	2.687 (3)	177 (4)
N3—H31⋯O2	0.90 (4)	2.20 (4)	2.873 (3)	132 (3)
O1—H1⋯O52^ii^	0.91 (4)	1.67 (4)	2.580 (3)	176 (4)

Symmetry codes: (i) 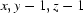; (ii) 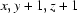.

**Fig. 1.**
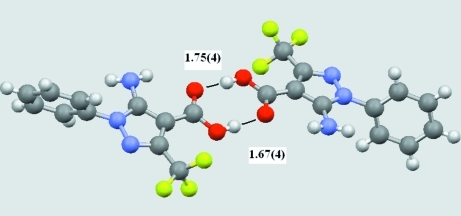
The asymetric unit of (I), showing the atom-numbering scheme. Displacement ellipsoids are drawn at the 50% probability level. H atoms are shown as small spheres of arbitrary radii and the intramolecular hydrogen bond is shown as dashed lines.

**Fig. 2.**
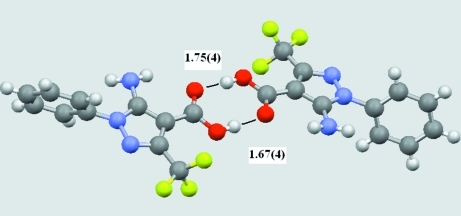
Part of the crystal structure of (I), showing the formation of a cyclic R22(8) pattern. [Symmetry code: (i) x, y + 1, z + 1].

## supplementary crystallographic information

### Comment

We report here the structure of the title compound (I), isolated during attempts to synthesize Ti(C_5_H_5_)_2_(C_11_H_7_F_3_N_3_O_2_)_2_. In the title compound C_11_H_8_F_3_N_3_O_2_, there are two molecules in the asymmetric unit that differ in the dihedral angle formed between the phenyl and pyrazole rings, 65.3 (2) and 85.6 (2)°. The pairs of molecules are held together by O—H···O hydrogen bonds between the carboxyl groups, forming a centrosymmetric dimer with an R^2^_2 _(8) motif, Fig. 2. (Bernstein *et al.*, 1995) . Related compounds of this ligand show antiproliferative and apoptotic effects against K562, K562-R (imatinib mesilate resistant), HL60 and multidrug resistant HL60 cell lines (Maggio *et al.*, 2008).

### Experimental

The synthesis of title compound has been described by Maggio *et al.*, 2008. The title compound was reacted with titanocene dichloride, (C_5_H_5_)_2_TiCl_2_. Elemental analysis {C, 53.50, H, 3.37, N, 11.70%. Found: C, 53.66, H, 3.45, N, 11.54%} indicated formation of Cp_2_Ti(ligand)_2_,= Ti(C_5_H_5_)_2_(C_11_H_7_F_3_N_3_O_2_)_2_, ligand = 5-amino-1-phenyl-3- (trifluoromethyl)-1*H*-pyrazole-4-carboxylate. Melting point 190–195°C. IR(nujol, cm-1): 3439w, 3332w (N—H), 3049w (Carom-H), 1661 s, 1611 s (COO), 1537 s, 1486 s (C=N + C=C). 1H NMR (CDCl_3_): 5.59br (10*H*, Ti—C_5_H_5_), 6.40br, 6.60br (6*H*, –NH_2_), 7.55 mbr (10*H*, Carom-H). Good solubility in chloroform, methanol, dimethylsulfoxide; lower solubilty in acetonitrile; insoluble in water. Upon recrystallization flat needles were obtained, they were studied to determine its crystal and molecular structure and revealed only the ligand. Ligand cleavage is a non unusual feature for Ti complexes that show a marked tendency to evolve towards titanium dioxide (Caruso & Rossi, 2004). The title compound synthesis has been described (Maggio *et al.*). Related compounds of this ligand show antiproliferative and apoptotic effects against K562, K562—*R* (imatinib mesilate resistant), HL60 and multidrug resistant HL60 cell lines (Maggio *et al.*, 2008).

### Refinement

H atoms bonded to C atoms were located from difference maps and treated as riding model, with C—H distance of 0.95 Å and with U_iso_(H) = 1.2U_eq_(C). O and N-bound H atoms were freely refined, giving distances and U_iso_ in the ranges O-H = [ 0.91 (4)-0.94 (4) ]Å, N-H = [0.85 (4)-0.95 (4)] Å and U_iso_(H) = [0.067 (12)-0.076 (14)] Å^2 ^U_eq_(O) and U_iso_(H) = [0.041 (9)-0.066 (12)] Å^2 ^U_eq_(N).

### Crystal data

**Table:**

C_11_H_8_F_3_N_3_O_2_	*F*(000) = 1104
*M**_r_* = 271.20	*D*_x_ = 1.619 Mg m^−^^3^
Monoclinic, *P*2_1_/*n*	Mo *K*α radiation, λ = 0.71073 Å
Hall symbol: -P 2yn	Cell parameters from 3829 reflections
*a* = 9.757 (3) Å	θ = 2.2–22.3°
*b* = 10.740 (3) Å	µ = 0.15 mm^−^^1^
*c* = 21.277 (6) Å	*T* = 125 K
β = 93.716 (3)°	Flat needle, pale yellow
*V* = 2225 (1) Å^3^	0.30 × 0.18 × 0.05 mm
*Z* = 8	

### Data collection

**Table:**

Bruker APEXII CCD diffractometer	3772 independent reflections
Radiation source: fine-focus sealed tube	2737 reflections with *I* > 2σ(*I*)
graphite	*R*_int_ = 0.072
φ and ω scans	θ_max_ = 24.7°, θ_min_ = 1.9°
Absorption correction: multi-scan (*SADABS*; Bruker, 1997)	*h* = −11→11
*T*_min_ = 0.957, *T*_max_ = 0.993	*k* = −12→12
20692 measured reflections	*l* = −25→24

### Refinement

**Table:**

Refinement on *F*^2^	Secondary atom site location: difference Fourier map
Least-squares matrix: full	Hydrogen site location: inferred from neighbouring sites
*R*[*F*^2^ > 2σ(*F*^2^)] = 0.051	H atoms treated by a mixture of independent and constrained refinement
*wR*(*F*^2^) = 0.140	*w* = 1/[σ^2^(*F*_o_^2^) + (0.0233*P*)^2^ + 1.9621*P*] where *P* = (*F*_o_^2^ + 2*F*_c_^2^)/3
*S* = 1.01	(Δ/σ)_max_ = 0.001
3772 reflections	Δρ_max_ = 0.43 e Å^−^^3^
368 parameters	Δρ_min_ = −0.32 e Å^−^^3^
0 restraints	Extinction correction: *SHELXL97* (Sheldrick, 2008), Fc^*^=kFc[1+0.001xFc^2^λ^3^/sin(2θ)]^-1/4^
Primary atom site location: structure-invariant direct methods	Extinction coefficient: 0.0099 (12)

### Special details

**Table:**

Geometry. All e.s.d.'s (except the e.s.d. in the dihedral angle between two l.s. planes) are estimated using the full covariance matrix. The cell e.s.d.'s are taken into account individually in the estimation of e.s.d.'s in distances, angles and torsion angles; correlations between e.s.d.'s in cell parameters are only used when they are defined by crystal symmetry. An approximate (isotropic) treatment of cell e.s.d.'s is used for estimating e.s.d.'s involving l.s. planes.
Refinement. Refinement of *F*^2^ against ALL reflections. The weighted *R*-factor *wR* and goodness of fit *S* are based on *F*^2^, conventional *R*-factors *R* are based on *F*, with *F* set to zero for negative *F*^2^. The threshold expression of *F*^2^ > σ(*F*^2^) is used only for calculating *R*-factors(gt) *etc*. and is not relevant to the choice of reflections for refinement. *R*-factors based on *F*^2^ are statistically about twice as large as those based on *F*, and *R*- factors based on ALL data will be even larger.

### Fractional atomic coordinates and isotropic or equivalent isotropic displacement parameters (Å^2^)

**Table:**

	*x*	*y*	*z*	*U*_iso_*/*U*_eq_	
F1	1.0029 (2)	0.57631 (18)	0.86748 (8)	0.0449 (5)	
F2	0.88145 (19)	0.61681 (17)	0.94547 (8)	0.0402 (5)	
F3	1.03719 (18)	0.74508 (17)	0.91981 (8)	0.0410 (5)	
F51	0.5059 (2)	0.47366 (17)	0.12944 (8)	0.0417 (5)	
F52	0.62681 (18)	0.41822 (16)	0.05332 (8)	0.0380 (5)	
F53	0.43613 (17)	0.32665 (16)	0.06739 (7)	0.0361 (5)	
O1	0.8071 (2)	0.8727 (2)	0.96933 (9)	0.0323 (5)	
O2	0.6443 (2)	0.98989 (19)	0.91963 (9)	0.0295 (5)	
O51	0.6194 (2)	0.1440 (2)	0.01774 (9)	0.0312 (5)	
O52	0.7542 (2)	0.00154 (19)	0.06715 (9)	0.0343 (5)	
N1	0.7329 (2)	0.7741 (2)	0.76427 (10)	0.0268 (6)	
N2	0.8258 (2)	0.6935 (2)	0.79385 (10)	0.0283 (6)	
N3	0.5911 (3)	0.9467 (2)	0.78704 (13)	0.0301 (6)	
N51	0.7186 (2)	0.2162 (2)	0.22548 (10)	0.0263 (6)	
N52	0.6454 (3)	0.3151 (2)	0.19957 (10)	0.0274 (6)	
N53	0.8148 (3)	0.0243 (3)	0.19747 (13)	0.0334 (7)	
C1	0.6874 (3)	0.7528 (3)	0.70017 (12)	0.0280 (7)	
C2	0.6161 (3)	0.6448 (3)	0.68442 (13)	0.0307 (7)	
H2	0.5971	0.5859	0.7160	0.037*	
C3	0.5730 (3)	0.6233 (3)	0.62231 (13)	0.0340 (8)	
H3	0.5244	0.5491	0.6111	0.041*	
C4	0.6001 (3)	0.7086 (3)	0.57672 (14)	0.0385 (8)	
H4	0.5702	0.6932	0.5340	0.046*	
C5	0.6705 (4)	0.8163 (3)	0.59258 (14)	0.0412 (8)	
H5	0.6886	0.8751	0.5608	0.049*	
C6	0.7154 (3)	0.8395 (3)	0.65493 (13)	0.0350 (8)	
H6	0.7643	0.9135	0.6661	0.042*	
C7	0.8382 (3)	0.7328 (3)	0.85250 (13)	0.0269 (7)	
C8	0.7540 (3)	0.8365 (3)	0.86272 (12)	0.0258 (7)	
C9	0.6881 (3)	0.8604 (3)	0.80423 (12)	0.0253 (6)	
C10	0.7303 (3)	0.9061 (3)	0.91922 (13)	0.0265 (7)	
C11	0.9388 (3)	0.6684 (3)	0.89644 (14)	0.0333 (7)	
C51	0.7711 (3)	0.2189 (3)	0.28986 (12)	0.0258 (7)	
C52	0.6897 (3)	0.1791 (3)	0.33664 (13)	0.0303 (7)	
H52	0.5997	0.1485	0.3264	0.036*	
C53	0.7412 (3)	0.1847 (3)	0.39859 (14)	0.0341 (7)	
H53	0.6865	0.1572	0.4313	0.041*	
C54	0.8717 (3)	0.2301 (3)	0.41322 (13)	0.0344 (8)	
H54	0.9060	0.2352	0.4560	0.041*	
C55	0.9524 (3)	0.2681 (3)	0.36596 (14)	0.0343 (7)	
H55	1.0429	0.2976	0.3761	0.041*	
C56	0.9021 (3)	0.2632 (3)	0.30386 (14)	0.0319 (7)	
H56	0.9570	0.2901	0.2712	0.038*	
C57	0.6225 (3)	0.2847 (3)	0.13997 (12)	0.0253 (6)	
C58	0.6793 (3)	0.1676 (3)	0.12543 (12)	0.0256 (7)	
C59	0.7408 (3)	0.1272 (3)	0.18279 (12)	0.0262 (7)	
C60	0.6867 (3)	0.0985 (3)	0.06813 (13)	0.0277 (7)	
C61	0.5484 (3)	0.3755 (3)	0.09757 (13)	0.0286 (7)	
H1	0.792 (4)	0.918 (4)	1.004 (2)	0.076 (14)*	
H30	0.597 (4)	0.987 (4)	0.748 (2)	0.066 (12)*	
H31	0.579 (4)	1.000 (3)	0.8190 (17)	0.047 (10)*	
H51	0.628 (4)	0.087 (4)	−0.0156 (19)	0.067 (12)*	
H530	0.830 (3)	0.001 (3)	0.2381 (17)	0.041 (9)*	
H531	0.807 (4)	−0.029 (3)	0.1682 (18)	0.047 (11)*	

### Atomic displacement parameters (Å^2^)

**Table:**

	*U*^11^	*U*^22^	*U*^33^	*U*^12^	*U*^13^	*U*^23^
F1	0.0527 (12)	0.0511 (12)	0.0302 (10)	0.0212 (10)	−0.0019 (8)	−0.0080 (8)
F2	0.0541 (12)	0.0421 (11)	0.0240 (9)	0.0044 (9)	0.0002 (8)	0.0061 (8)
F3	0.0383 (11)	0.0534 (12)	0.0302 (10)	−0.0014 (9)	−0.0061 (8)	−0.0047 (8)
F51	0.0600 (12)	0.0355 (11)	0.0290 (10)	0.0142 (9)	−0.0017 (9)	−0.0026 (8)
F52	0.0473 (11)	0.0392 (11)	0.0280 (9)	−0.0021 (9)	0.0055 (8)	0.0098 (8)
F53	0.0368 (10)	0.0450 (11)	0.0255 (9)	0.0005 (8)	−0.0054 (8)	0.0025 (8)
O1	0.0468 (13)	0.0355 (13)	0.0141 (10)	0.0050 (10)	−0.0018 (9)	−0.0034 (9)
O2	0.0381 (12)	0.0312 (12)	0.0191 (10)	0.0040 (10)	0.0009 (8)	−0.0016 (8)
O51	0.0446 (13)	0.0341 (12)	0.0145 (10)	0.0059 (10)	−0.0013 (9)	−0.0016 (9)
O52	0.0547 (14)	0.0304 (12)	0.0171 (10)	0.0095 (11)	−0.0016 (9)	−0.0027 (8)
N1	0.0323 (14)	0.0339 (14)	0.0140 (11)	0.0015 (11)	−0.0012 (10)	−0.0020 (10)
N2	0.0310 (14)	0.0353 (15)	0.0184 (12)	0.0013 (11)	−0.0004 (10)	−0.0021 (10)
N3	0.0386 (15)	0.0329 (15)	0.0187 (13)	0.0037 (12)	0.0008 (11)	−0.0015 (12)
N51	0.0364 (14)	0.0264 (13)	0.0158 (12)	0.0009 (11)	−0.0012 (10)	−0.0018 (10)
N52	0.0363 (14)	0.0285 (14)	0.0169 (12)	0.0022 (11)	−0.0016 (10)	−0.0004 (10)
N53	0.0501 (17)	0.0324 (16)	0.0170 (14)	0.0067 (13)	−0.0026 (12)	−0.0009 (12)
C1	0.0320 (16)	0.0357 (18)	0.0162 (14)	0.0050 (14)	0.0012 (12)	−0.0017 (12)
C2	0.0316 (16)	0.0389 (19)	0.0212 (15)	0.0045 (14)	−0.0010 (12)	−0.0017 (13)
C3	0.0328 (17)	0.042 (2)	0.0268 (16)	0.0037 (15)	−0.0016 (13)	−0.0096 (14)
C4	0.0422 (19)	0.051 (2)	0.0219 (16)	0.0123 (16)	−0.0039 (14)	−0.0082 (15)
C5	0.052 (2)	0.050 (2)	0.0224 (16)	0.0114 (17)	0.0047 (14)	0.0059 (15)
C6	0.0403 (18)	0.0407 (19)	0.0243 (16)	0.0009 (15)	0.0036 (13)	−0.0014 (14)
C7	0.0329 (16)	0.0304 (17)	0.0172 (14)	−0.0030 (13)	0.0007 (12)	0.0010 (12)
C8	0.0346 (16)	0.0274 (16)	0.0153 (14)	−0.0040 (13)	0.0015 (12)	−0.0009 (12)
C9	0.0300 (16)	0.0262 (16)	0.0200 (14)	−0.0032 (13)	0.0029 (12)	−0.0019 (12)
C10	0.0352 (17)	0.0258 (16)	0.0183 (14)	−0.0067 (14)	0.0000 (12)	0.0012 (12)
C11	0.0389 (18)	0.0370 (18)	0.0236 (16)	0.0012 (15)	−0.0002 (13)	−0.0021 (14)
C51	0.0333 (16)	0.0267 (16)	0.0169 (14)	0.0001 (13)	−0.0028 (12)	−0.0021 (12)
C52	0.0317 (16)	0.0367 (18)	0.0227 (15)	0.0003 (14)	0.0030 (12)	0.0007 (13)
C53	0.0415 (19)	0.0408 (19)	0.0205 (15)	0.0057 (15)	0.0055 (13)	0.0016 (13)
C54	0.0443 (19)	0.0393 (19)	0.0185 (15)	0.0069 (15)	−0.0074 (13)	−0.0042 (13)
C55	0.0364 (18)	0.0350 (19)	0.0307 (17)	−0.0008 (14)	−0.0047 (14)	−0.0044 (14)
C56	0.0370 (18)	0.0348 (18)	0.0242 (16)	−0.0050 (14)	0.0049 (13)	−0.0027 (13)
C57	0.0336 (16)	0.0254 (16)	0.0168 (14)	−0.0027 (13)	0.0006 (12)	−0.0002 (12)
C58	0.0360 (17)	0.0243 (16)	0.0164 (14)	−0.0035 (13)	0.0021 (12)	−0.0008 (11)
C59	0.0318 (16)	0.0280 (17)	0.0191 (14)	−0.0014 (13)	0.0036 (12)	−0.0015 (12)
C60	0.0362 (17)	0.0297 (18)	0.0170 (15)	−0.0045 (14)	0.0001 (12)	−0.0013 (12)
C61	0.0359 (17)	0.0289 (17)	0.0209 (15)	0.0004 (13)	0.0015 (13)	−0.0048 (12)

### Geometric parameters (Å, °)

**Table:**

F1—C11	1.341 (3)	C2—C3	1.380 (4)
F2—C11	1.336 (3)	C2—H2	0.9500
F3—C11	1.336 (4)	C3—C4	1.372 (5)
F51—C61	1.335 (3)	C3—H3	0.9500
F52—C61	1.333 (3)	C4—C5	1.376 (5)
F53—C61	1.340 (3)	C4—H4	0.9500
O1—C10	1.313 (3)	C5—C6	1.392 (4)
O1—H1	0.91 (4)	C5—H5	0.9500
O2—C10	1.231 (3)	C6—H6	0.9500
O51—C60	1.315 (3)	C7—C8	1.410 (4)
O51—H51	0.94 (4)	C7—C11	1.482 (4)
O52—C60	1.232 (4)	C8—C9	1.387 (4)
N1—C9	1.350 (4)	C8—C10	1.447 (4)
N1—N2	1.376 (3)	C51—C56	1.379 (4)
N1—C1	1.425 (3)	C51—C52	1.380 (4)
N2—C7	1.316 (4)	C52—C53	1.381 (4)
N3—C9	1.358 (4)	C52—H52	0.9500
N3—H30	0.95 (4)	C53—C54	1.381 (5)
N3—H31	0.90 (4)	C53—H53	0.9500
N51—C59	1.346 (4)	C54—C55	1.378 (4)
N51—N52	1.376 (3)	C54—H54	0.9500
N51—C51	1.431 (3)	C55—C56	1.380 (4)
N52—C57	1.315 (3)	C55—H55	0.9500
N53—C59	1.346 (4)	C56—H56	0.9500
N53—H530	0.90 (3)	C57—C58	1.416 (4)
N53—H531	0.85 (4)	C57—C61	1.484 (4)
C1—C6	1.379 (4)	C58—C59	1.393 (4)
C1—C2	1.383 (4)	C58—C60	1.433 (4)
			
C10—O1—H1	114 (3)	O2—C10—C8	121.9 (3)
C60—O51—H51	108 (2)	O1—C10—C8	114.9 (3)
C9—N1—N2	112.0 (2)	F2—C11—F3	107.0 (2)
C9—N1—C1	128.2 (2)	F2—C11—F1	106.6 (2)
N2—N1—C1	119.5 (2)	F3—C11—F1	106.3 (2)
C7—N2—N1	104.4 (2)	F2—C11—C7	113.1 (3)
C9—N3—H30	118 (2)	F3—C11—C7	112.2 (3)
C9—N3—H31	110 (2)	F1—C11—C7	111.3 (2)
H30—N3—H31	114 (3)	C56—C51—C52	121.3 (3)
C59—N51—N52	112.3 (2)	C56—C51—N51	118.9 (3)
C59—N51—C51	126.7 (2)	C52—C51—N51	119.8 (3)
N52—N51—C51	120.8 (2)	C51—C52—C53	119.0 (3)
C57—N52—N51	104.2 (2)	C51—C52—H52	120.5
C59—N53—H530	120 (2)	C53—C52—H52	120.5
C59—N53—H531	111 (2)	C54—C53—C52	120.2 (3)
H530—N53—H531	121 (3)	C54—C53—H53	119.9
C6—C1—C2	121.1 (3)	C52—C53—H53	119.9
C6—C1—N1	119.8 (3)	C55—C54—C53	120.2 (3)
C2—C1—N1	119.1 (3)	C55—C54—H54	119.9
C3—C2—C1	119.3 (3)	C53—C54—H54	119.9
C3—C2—H2	120.3	C54—C55—C56	120.2 (3)
C1—C2—H2	120.3	C54—C55—H55	119.9
C4—C3—C2	120.3 (3)	C56—C55—H55	119.9
C4—C3—H3	119.9	C51—C56—C55	119.2 (3)
C2—C3—H3	119.9	C51—C56—H56	120.4
C3—C4—C5	120.3 (3)	C55—C56—H56	120.4
C3—C4—H4	119.9	N52—C57—C58	112.8 (2)
C5—C4—H4	119.9	N52—C57—C61	117.9 (2)
C4—C5—C6	120.3 (3)	C58—C57—C61	129.2 (2)
C4—C5—H5	119.8	C59—C58—C57	103.7 (2)
C6—C5—H5	119.8	C59—C58—C60	122.8 (3)
C1—C6—C5	118.7 (3)	C57—C58—C60	133.5 (3)
C1—C6—H6	120.6	N51—C59—N53	122.3 (3)
C5—C6—H6	120.6	N51—C59—C58	107.0 (2)
N2—C7—C8	112.4 (2)	N53—C59—C58	130.7 (3)
N2—C7—C11	117.7 (3)	O52—C60—O51	122.6 (3)
C8—C7—C11	129.8 (3)	O52—C60—C58	120.5 (3)
C9—C8—C7	104.4 (2)	O51—C60—C58	116.8 (3)
C9—C8—C10	124.1 (3)	F52—C61—F51	107.4 (2)
C7—C8—C10	131.5 (3)	F52—C61—F53	106.6 (2)
N1—C9—N3	123.2 (3)	F51—C61—F53	106.3 (2)
N1—C9—C8	106.7 (2)	F52—C61—C57	112.1 (2)
N3—C9—C8	130.0 (3)	F51—C61—C57	111.5 (2)
O2—C10—O1	123.2 (3)	F53—C61—C57	112.7 (2)
			
C9—N1—N2—C7	−0.7 (3)	N2—C7—C11—F1	−0.1 (4)
C1—N1—N2—C7	−175.4 (2)	C8—C7—C11—F1	−177.2 (3)
C59—N51—N52—C57	0.2 (3)	C59—N51—C51—C56	83.1 (4)
C51—N51—N52—C57	175.8 (2)	N52—N51—C51—C56	−91.7 (3)
C9—N1—C1—C6	69.0 (4)	C59—N51—C51—C52	−97.9 (4)
N2—N1—C1—C6	−117.2 (3)	N52—N51—C51—C52	87.3 (3)
C9—N1—C1—C2	−111.3 (3)	C56—C51—C52—C53	0.3 (5)
N2—N1—C1—C2	62.5 (4)	N51—C51—C52—C53	−178.7 (3)
C6—C1—C2—C3	0.3 (4)	C51—C52—C53—C54	0.4 (5)
N1—C1—C2—C3	−179.4 (3)	C52—C53—C54—C55	−1.2 (5)
C1—C2—C3—C4	−0.3 (4)	C53—C54—C55—C56	1.3 (5)
C2—C3—C4—C5	0.0 (5)	C52—C51—C56—C55	−0.1 (5)
C3—C4—C5—C6	0.3 (5)	N51—C51—C56—C55	178.9 (3)
C2—C1—C6—C5	0.0 (5)	C54—C55—C56—C51	−0.6 (5)
N1—C1—C6—C5	179.6 (3)	N51—N52—C57—C58	−0.2 (3)
C4—C5—C6—C1	−0.3 (5)	N51—N52—C57—C61	−177.9 (2)
N1—N2—C7—C8	0.9 (3)	N52—C57—C58—C59	0.1 (3)
N1—N2—C7—C11	−176.6 (2)	C61—C57—C58—C59	177.5 (3)
N2—C7—C8—C9	−0.8 (3)	N52—C57—C58—C60	−176.8 (3)
C11—C7—C8—C9	176.3 (3)	C61—C57—C58—C60	0.6 (5)
N2—C7—C8—C10	177.1 (3)	N52—N51—C59—N53	177.5 (3)
C11—C7—C8—C10	−5.7 (5)	C51—N51—C59—N53	2.2 (5)
N2—N1—C9—N3	−176.8 (3)	N52—N51—C59—C58	−0.2 (3)
C1—N1—C9—N3	−2.6 (5)	C51—N51—C59—C58	−175.4 (3)
N2—N1—C9—C8	0.2 (3)	C57—C58—C59—N51	0.1 (3)
C1—N1—C9—C8	174.3 (3)	C60—C58—C59—N51	177.4 (3)
C7—C8—C9—N1	0.4 (3)	C57—C58—C59—N53	−177.3 (3)
C10—C8—C9—N1	−177.8 (3)	C60—C58—C59—N53	0.0 (5)
C7—C8—C9—N3	177.0 (3)	C59—C58—C60—O52	−3.2 (5)
C10—C8—C9—N3	−1.1 (5)	C57—C58—C60—O52	173.2 (3)
C9—C8—C10—O2	2.9 (5)	C59—C58—C60—O51	177.1 (3)
C7—C8—C10—O2	−174.7 (3)	C57—C58—C60—O51	−6.4 (5)
C9—C8—C10—O1	−177.2 (3)	N52—C57—C61—F52	115.3 (3)
C7—C8—C10—O1	5.2 (5)	C58—C57—C61—F52	−62.0 (4)
N2—C7—C11—F2	−120.1 (3)	N52—C57—C61—F51	−5.1 (4)
C8—C7—C11—F2	62.9 (4)	C58—C57—C61—F51	177.6 (3)
N2—C7—C11—F3	118.8 (3)	N52—C57—C61—F53	−124.5 (3)
C8—C7—C11—F3	−58.2 (4)	C58—C57—C61—F53	58.2 (4)

### Hydrogen-bond geometry (Å, °)

**Table:**

*D*—H···*A*	*D*—H	H···*A*	*D*···*A*	*D*—H···*A*
N53—H531···O52	0.85 (4)	2.20 (4)	2.808 (3)	128 (3)
O51—H51···O2^i^	0.94 (4)	1.75 (4)	2.687 (3)	177 (4)
N3—H31···O2	0.90 (4)	2.20 (4)	2.873 (3)	132 (3)
O1—H1···O52^ii^	0.91 (4)	1.67 (4)	2.580 (3)	176 (4)

Symmetry codes: (i) *x*, *y*−1, *z*−1; (ii) *x*, *y*+1, *z*+1.
